# A Novel Rice Curl Dwarf-Associated Picornavirus Encodes a 3C Serine Protease Recognizing Uncommon EPT/S Cleavage Sites

**DOI:** 10.3389/fmicb.2021.757451

**Published:** 2021-10-13

**Authors:** Tianze Zhang, Chenyang Li, Mengji Cao, Dan Wang, Qi Wang, Yi Xie, Shibo Gao, Shuai Fu, Xueping Zhou, Jianxiang Wu

**Affiliations:** ^1^State Key Laboratory of Rice Biology, Institute of Biotechnology, Zhejiang University, Hangzhou, China; ^2^National Citrus Engineering and Technology Research Center, Citrus Research Institute, Southwest University, Beibei, China; ^3^State Key Laboratory for Biology of Plant Diseases and Insect Pests, Institute of Plant Protection, Chinese Academy of Agricultural Sciences, Beijing, China; ^4^Hainan Research Institute of Zhejiang University, Hainan, China

**Keywords:** RNA-seq, rice curl dwarf-associated virus, picornavirus, 3C protease, serine protease, cleavage site

## Abstract

Picornaviruses cause diseases in a wide range of vertebrates, invertebrates and plants. Here, a novel picornavirus was identified by RNA-seq technology from rice plants showing dwarfing and curling symptoms, and the name rice curl dwarf-associated virus (RCDaV) is tentatively proposed. The RCDaV genome consists of an 8,987 nt positive-stranded RNA molecule, excluding a poly(A) tail, that encodes two large polyproteins. Using *in vitro* cleavage assays, we have identified that the RCDaV 3C protease (3Cpro) as a serine protease recognizes the conserved EPT/S cleavage site which differs from the classic Q(E)/G(S) sites cleaved by most picornaviral 3C chymotrypsin-like cysteine proteases. Therefore, we comprehensively deciphered the RCDaV genome organization and showed that the two polyproteins of RCDaV can be cleaved into 12 mature proteins. We found that seven unclassified picornaviruses also encode a 3Cpro similar to RCDaV, and use the highly conserved EPT/S as the cleavage site. The precise genome organizations of these viruses were illustrated. Moreover, RCDaV and the seven unclassified picornaviruses share high sequence identities and similar genome organizations, and cluster into a distinct clade in the order *Picornavirales*. Our study provides valuable information for the understanding of picornaviral 3Cpros, deciphers the genome organization of a few relatively obscure picornaviruses, and lays the foundation for further pathogenesis research on these viruses.

## Introduction

According to the 2020 taxonomic classification ratified by the International Committee on Taxonomy of Viruses (ICTV)^1^, the order *Picornavirales* contains eight officially classified families: *Picornaviridae*, *Dicistroviridae*, *Iflaviridae, Marnaviridae*, *Polycipiviridae*, *Secoviridae*, *Caliciviridae*, and *Solinviviridae*, with one subfamily, 103 genera, three subgenera, and a total of 323 species as well as an unassigned group containing unclassified picornaviruses^2,3^. Viruses in the order *Picornavirales* have positive sense RNA genomes with 5′-bound VPg (viral protein genome-linked) and 3′-poly(A) encapsidated in spherical viral particles about 30 nm in diameter. They infect vertebrates, arthropods, plants, fungi and algae (Sanfaçon et al., 2009). Their ORFs encode polyproteins, in which the non-structural region contains a typical “replication block” with a type III helicase, a chymotrypsin-like fold (cysteine) protease and a type I RNA-dependent RNA polymerase (Hel–Pro–Pol) (Le Gall et al., 2008).

Genome organizations vary significantly among picornaviruses. The genome of viruses in the family *Marnaviridae* or *Dicistroviridae* is dicistronic, while viruses in the families *Picornaviridae* and *Iflaviridae* possess a monocistronic genome, except the dicistronic viruses in the genus *Dicipivirus* (Yinda et al., 2017). Viruses in the family *Polycipiviridae* are polycistronic, with four or more consecutive ORFs in the 5′-proximal region and a long ORF encoding non-structural proteins in the 3′-proximal region (Olendraite et al., 2017). Viruses in the family *Secoviridae* possess either monopartite or bipartite genomes (Sanfaçon et al., 2009). It is noteworthy that plant-infecting picornaviruses are all classified in the family *Secoviridae* (Sanfaçon et al., 2009). Genomes of the viruses in the family *Caliciviridae* are not monocistronic but contain one or two additional ORFs (Le Gall et al., 2008). The capsid proteins of the viruses in the newly established family *Solinviviridae* can be expressed from subgenomic or genomic RNAs as an extension of the “replication block” (Brown et al., 2019). However, some picornaviruses have not been well classified yet as a result of divergent genome organizations, low sequence identities, as well as distant evolutionary relationships with viruses from the eight officially classified families, which are still referred to as unclassified picornaviruses.

Picornaviral polyproteins are post-translationally cleaved by virus-encoded 3C protease (3Cpro) and the leader protein (L) in a proteolytic manner, or co-translationally processed by the 2A protein via a non-proteolytic mechanism (Kjær and Belsham, 2018). The 3Cpro is responsible for most of the cleavage activities (Yang et al., 2017). Typical picornaviral 3Cpros are intermediates between the chymotrypsin-like serine proteases and the papain-like cysteine proteases (Sárkány and Polgár, 2003). Analysis of the crystal structure of picornaviral 3Cpro reveals its similarity with chymotrypsin-like serine protease (Allaire et al., 1994; Matthews et al., 1994), but a conserved cysteine (C) nucleophile replaces serine (S), resulting in a GxCG core motif instead of a GxSG core motif. Therefore, the classic catalytic triad in picornaviral 3Cpros contains a histidine (H), an aspartate/glutamate (D/E), and a conserved cysteine (Dessens and Lomonossoff, 1991; Sárkány and Polgár, 2003). However, the 3Cpros of the viruses in the family *Polycipiviridae* and several viruses in the family *Secoviridae* and *Marnaviridae* retain catalytic serine, indicating they encode the typical serine proteases (Olendraite et al., 2017; Mann and Sanfaçon, 2019).

Early studies have demonstrated the proteolytic activity of viral 3C protease using a series of *cis-* and *trans-*cleavage assays in rabbit reticulocyte cell-free transcription/translation system (Wetzel et al., 2013; Mann et al., 2017). In addition, the cleavage activity of 3Cpro encoded by *Ectropis obliqua* picorna-like *virus* (EOV) or rice tungro spherical virus (RTSV) has been identified using purified recombinant proteins expressed by *Escherichia coli* cells (Thole and Hull, 1998; Ye et al., 2012). The highly specific cleavage sites recognized by picornaviral 3Cpros are commonly Q/G, Q/S, and E/G dipeptides with specific amino acids (aa) flanking the cleavage sites (Seipelt et al., 1999; Sanfaçon, 2015), and it has been suggested that for the picornaviruses, the conserved His (H) residue in the S1 position of the substrate binding pocket (SBP) may be important for cleavage site recognition (Bazan and Fletterick, 1988; Mann and Sanfaçon, 2019). As 3Cpro plays a central role in polyprotein processing, classic picornaviral 3C cysteine proteases have been extensively studied in several families. However, research on the function and the cleavage sites recognition specificity of picornaviral serine 3Cpro remains scanty.

With the rapid development of high-throughput RNA-seq, a large number of novel viruses have been discovered in recent years (Massart et al., 2014; Shi et al., 2016). For example, Shi et al. (2016) discovered 1,445 novel RNA viruses in over 220 invertebrate species through deep transcriptome sequencing. In 2017, rice plants showing dwarfing and curled tillers were collected in paddy fields in the Zhejiang Province, China. Analysis of the sequencing data by RNA-seq allowed us to identify a new picornavirus, which was tentatively named rice curl dwarf-associated virus (RCDaV). Phylogenetic analysis showed that RCDaV and seven unclassified picornaviruses cluster into a distinct clade in the order *Picornavirales*, and encode a chymotrypsin-like serine protease with the conserved EPT/S cleavage sites.

## Materials and Methods

### Virus Source and Electron Microscopy

Rice plants showing dwarfing and curling symptoms were collected in 2017 from rice fields in the Zhejiang Province, China. For transmission electron microscope (TEM), the collected samples were homogenized in sterile deionized water (1 g tissue/5 mL water). After 5 min centrifugation at 5,000 × *g*, the supernatant was examined under a TEM (JEOL JEM-1010, Tokyo, Japan).

### RNA Sequencing and *de novo* Assembly

Total RNA was extracted from rice tissues using TRIzol reagent (Invitrogen, Carlsbad, United States). Total RNA (3 μg) was used for cDNA library constructions by the Zhejiang TianKe High-Technology Development Co., Ltd. (Hangzhou, China), and sequenced using the Illumina Hiseq^TM^ 4000 sequencing system (Illumina, San Diego, United States) as described previously (Zhang et al., 2020). The raw sequencing data was processed after removing low quality reads using the CLC Genomics Workbench 9.5 (Qiagen, Valencixa, United States). The resulting high quality reads were then mapped to the rice genome^4^ and the reads matched the rice genome were removed. The non-rice reads were then imported to the Trinity program for *de novo* assembly (Grabherr et al., 2011), and the assembled contigs were then subjected to BLASTx and BLASTp searches in the NCBI databases. The identified virus-like sequences were extracted according to the annotation information.

### RT-PCR, and 3′ and 5′ Rapid Amplification of the cDNA Ends-PCR

The 3′ and 5′ ends of the viral genomic RNA were obtained through rapid amplification of the cDNA ends (RACE) using a SMARTer RACE cDNA amplification kit as instructed (Clontech, Mountain View, United States). The resulting sequences were checked manually and then assembled to produce the final viral genome sequence. The complete viral genome sequence was confirmed by re-sequencing several fragments amplified by RT-PCR using specific primers (Supplementary Table 1). The conserved domains in the viral genome sequence were identified using the Conserved Domain Search Service (CD-Search) at the NCBI web server^5^ (Marchler-Bauer et al., 2015).

### RT-PCR Detection

Total RNA was isolated from rice plants using TRIzol reagent. the cDNA was generated from total RNA by reverse transcription using ReverTra Ace qPCR RT Master Mix with gDNA Remover (TOYOBO, Osaka, Japan) following the recommended protocol. The PCR was set up using the Green Taq Mix (Vazyme, Nanjing, China) according to the manufacturer’s instructions. Primer pair RCDaV-detection-F1/R1 (Supplementary Table 1) was used to detect RCDaV. PCR reaction (20 μL each) contained 10 μL 2 × Green Taq mix (Vazyme), 1 μL for each primer (10 μmol/L each), 1 μL cDNA and 7 μL sterile deionized water. Thermal cycles settings were 94°C for 2 min; 30 cycles of 94°C for 30 s, 53°C for 30 s, and 72°C for 10 s; and the final extension was 72°C for 10 min.

### Plasmid Construction

Protein expression vector pET-32a was modified by replacing the 6 × His tags at C-terminus with a 3 × FLAG tag. To construct a pET-32a-3 × FLAG vector, the original pET-32a vector was linearized via double digestion with *Not*I and *Xho*I restriction enzymes (Thermo Fisher Scientific, Waltham, United States). A 3 × FLAG tag containing a stop codon was fused to vector using the ClonExpress II one step cloning kit (Vazyme).

The substrate segments were PCR-amplified, and purified using a DNA gel extraction kit (Corning Life Sciences, Lowell, United States). Vector pET-32a-3 × FLAG was linearized through double digestion with *Bam*HI and *Sal*I restriction enzymes (Thermo Fisher Scientific). The purified PCR product and the linearized vector were fused using ClonExpress II one step cloning kit (Vazyme) according to the manufacturer’s instructions.

The protease segments (RCDaV-Pro_1054–1496_, MaPV-Pro_1094–1490_, ApGlV1-Pro_956–1355_), MBP tag, and SUMO tag with a stop codon were PCR-amplified. The original pET-28a vector was linearized through backward PCR-amplification to remove its tags, except the C-terminal His tag. Then, the purified PCR products were fused with the linearized vector using ClonExpress MultiS one step cloning kit (Vazyme). The plasmid for expressing tag-free 3Cpro was generated through fusing PCR-amplified *3Cpro* with the *Nco*I/*Xho*I linearized pET-28a vector using the ClonExpress II one step cloning kit (Vazyme). Primers used in this section are listed in Supplementary Table 1, details of the constructs are listed in Supplementary Table 2.

### Site-Directed Mutagenesis

The site-directed mutations were introduced to the gene segments by using Mut Express II Fast Mutagenesis Kit (Vazyme) according to the manufacturer’s instructions. The plasmids containing substrates or 3Cpros were backward PCR-amplified using primers carrying specific mutations of the codons, and then the purified PCR product was self-ligated. Primers used in this section are also listed in Supplementary Table 1, details of the mutated constructs are listed in Supplementary Table 2.

### *In vitro* Cleavage Assays

Substrates and 3Cpro proteins were prepared using a modified *E. coli* cell extract-based cell free protein expression kit (GZL Bioscience, Hangzhou, China). Briefly, the pET-32a and pET-28a vectors with desired gene sequences were individually amplified through PCR to generate the DNA templates. The resulting templates (15 μL/template) was used in a 35 μL reaction mixture composed of 17 μL reaction buffer, 13 μL *E. coli* cell extract, and 5 μL ddH_2_O. The mixtures were incubated at 30°C for 3 h for protein expression.

For *in vitro cis*-cleavage assay, after 3 h protein expression, the incubated reaction mixtures were individually mixed with 2 × SDS-PAGE sample buffer, boiled for 5 min, and then subjected to western blot analysis. For *in vitro trans*-cleavage assay, the protein expression incubation time was reduced to 1.5 h at 30°C. The resulting substrate and 3Cpro were mixed, and then incubated at 28°C for 1.5 h followed by western blot analysis.

### Prokaryotic Protein Expression and N-Terminal Edman Degradation Sequencing

FLAG-tagged fusion proteins were expressed with pET32a-3 × FLAG vector in *E. coli* strain BL21 (DE3) and purified with FLAG-beads followed by 12% SDS-PAGE. The cleavage protein bands were cut out and subjected to N-terminal Edman degradation sequencing by Tailian biotech Co., Ltd. (Beijing, China).

### Western Blot

Western blot analyses were conducted as previously described (Fu et al., 2018) with an anti-FLAG or anti-6 × His murine antibody.

### Phylogenetic Analyses

Sequences of viruses in different families in the order *Picornavirales* and the unassigned members were retrieved from the GenBank database (Supplementary Table 3). The deduced amino acid sequences of RdRPs and 3Cpros were aligned using the Muscle v 3.8.31 (Edgar, 2004) and the phylogeny analyses were performed using the MEGA X software (Kumar et al., 2018) via the maximum-likelihood method based on the JTT matrix-based model with a bootstrap of 1000 replications (Felsenstein, 1985; Jones et al., 1992). The resulting phylogeny trees were presented using the iTOL online tool^6^ (Letunic and Bork, 2019).

### Cleavage Site Conservation Analyses and Sequence Logo Generation

The sequence of cleavage sites (P4–P4′) in RCDaV and the seven unclassified members were extracted and analyzed using the TBtools software (Chen et al., 2020). The sequence logos were generated with the same software to demonstrate the conservation of cleavage sites.

### Gene Synthesis

*MaPV-P2*_1–310_ (5,033–5,962 nt) and *MaPV-Pro*_1094–1490_ (1,493–2,683 nt) of maize-associated picornavirus (MaPV, accession number MF425855) and *ApGlV1-P2*_1–199_ (3,196–3,792 nt) and *ApGlV1-Pro*_956–1355_ (6,061–7,260 nt) of aphis glycines virus 1 (ApGlV1, accession number KM015260) were synthesized by the GenScript Biotech (Nanjing, China).

### Data Availability

The genome sequence of RCDaV has been deposited in GenBank as accession number MW725267.

## Results

### Identification of a Novel Rice-Associated Picornavirus

**FIGURE 1:**
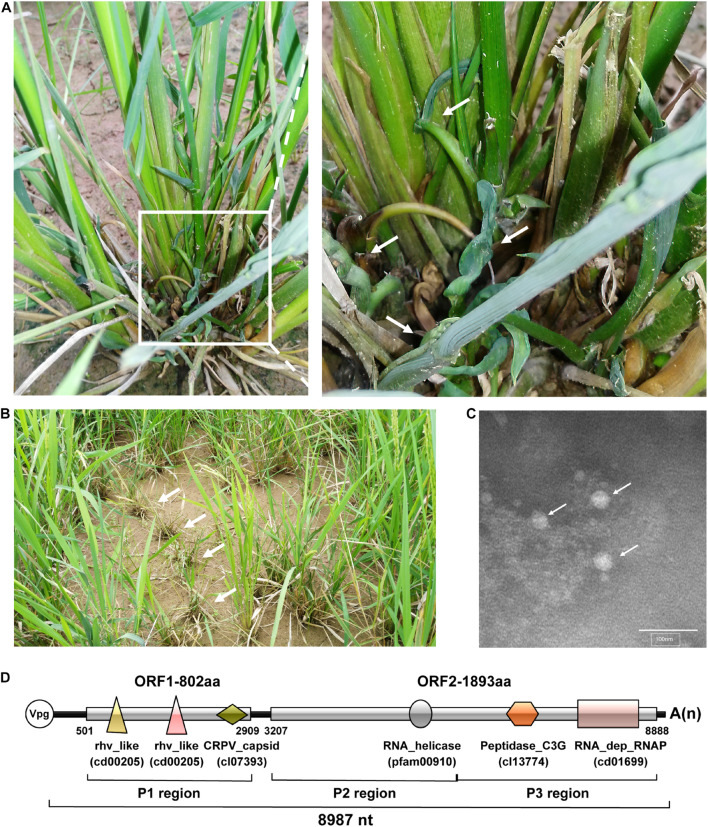
Rice plant symptoms, virions, and genome organization of rice curl dwarf-associated picornavirus (RCDaV). **(A)** The rice plants showing curling and dwarfing symptoms. The right image is the enlargement of the box in the left image. White arrows indicate curled tilters. **(B)** The rice plants showing dwarfing in the field. White arrows indicate symptomatic rice plants. **(C)** Electron micrograph of putative RCDaV virions. Extracts were prepared from curled tilters of rice and examined under a transmission electron microscope after negative staining with a 2% phosphotungstic acid solution, pH 7.0. Bar = 100 nm. White arrows indicate viral particle-like structures. **(D)** A schematic diagram of RCDaV genome organization. Three protein regions as well as six predicted domains are shown below the diagram. The six different domains are represented by different shapes and colors, and the name of the domains are marked below the shapes. The VPg is a viral protein covalently linked to the 5′ end of the viral genomic RNA.

During a field survey in the Zhejiang Province, China in August 2017, rice plants showing dwarfing and curling symptoms were observed and sampled (Figures 1A,B). Known rice-infecting viruses (i.e., rice black-streaked dwarf virus, southern rice black-streaked dwarf virus, rice stripe virus, rice ragged stunt virus, rice dwarf virus, rice gall dwarf virus, rice grassy stunt virus, rice stripe mosaic virus) failed to be detected in these rice plants by Dot-ELISA or RT-PCR (data not shown). However, electron microscopy analysis showed the presence of non-enveloped spherical particles with a diameter of approximately 30 nm in the rice plant crude extract (Figure 1C). To investigate the nature of these virus-like particles, we extracted total RNA from collected rice samples and analyzed them through RNA-seq. The RNA-seq analysis produced a total of 86,216,078 clean reads, and 77,020,091 of them (89.33%) were mapped to the reference rice genome and thus removed. A total of 9,195,987 clean reads were assembled to produce 63,682 contigs with 200–14,135 nt in length. Among these reads, 118,000 were further assembled into a long contig of 8,675 nt, and the BLASTx result revealed that this contig may represent a novel picornavirus. Then, the full-length sequence of this putative viral RNA genome was obtained by RT-PCR, 5′ and 3′UTR RACEs, revealing an 8,987 nt positive-stranded RNA genome, excluding a poly(A) tail (Figure 1D). This viral genome contains two large ORFs: a 2,409 nt ORF encodes an 802 aa protein (thereafter referred to as ORF1) and a 5,682 nt ORF encodes an 1,893 aa protein (ORF2). These two ORFs are separated by a 297 nt intergenic region (IGR). The complete genome sequence has been submitted to GenBank under the accession number MW725267.

Using the NCBI Conserved Domain Database (CDD) tool, we identified three conserved capsid protein (CP) domains in ORF1, including two rhv-like domains (cd00205, aa 56–235, and aa 343–529) and a cricket paralysis virus (CrPV) capsid-like domain (cl07393, aa 636-769) (Figure 1D). The conserved non-structural protein domains in ORF2 include an RNA helicase domain (pfam00910, aa 678–788), a Peptidase_C3G domain (cl13774, aa 1088-1310), and an RNA_dep_RNAP domain (cd01699, aa 1,507–1,804) (Figure 1D). The BLASTp search reveals that the RNA_dep_RNAP domain shares the closest aa sequence identity (90.6%) with the polyprotein of maize-associated picornavirus (MaPV) (AUH27292.1) and 65–90% aa sequence identities with several unclassified picornaviruses such as *Tetranychus urticae*-associated picorna-like virus 1 (TUaPV1) (QIN54759.1), aphis glycines virus 1 (ApGlV1) (AHC72013.1) and cherry virus Trakiya (CVT) (YP_009551963.1) (Supplementary Table 4). According to these results, we propose this virus as a novel species in the order *Picornavirales*, and tentatively name it rice curl dwarf-associated virus (RCDaV). Analysis of the sampled rice plants through RT-PCR showed that RCDaV was present in rice leaves, stems, and roots (Supplementary Figure 1). RCDaV was also detected in barnyard grass plants with similar dwarfing and curling symptoms, which were collected in the same rice field (Supplementary Figure 1).

The genome of most picornaviruses is a single-stranded positive-sense RNA, and lacks a 5′ cap structure needed for the initiation of protein synthesis. Instead, a small viral protein 3B (also known as VPg) is covalently linked to the 5′ end of the viral genomic RNA (Le Gall et al., 2008; Sanfaçon et al., 2009). Thus, we postulate that RCDaV also follows this rule, like other picornaviruses. Moreover, the predicted AUG translation initiation codons for the two polyproteins are set in Kozak consensus sequences (AAAA_501_UGG, ATCA_3207_UGG) (Kozak, 1999).

In this study, we adopted the L344 nomenclature system (Rueckert and Wimmer, 1984) to name RCDaV proteins because the arrangement of RCDaV proteins is similar to that of canine picodicistrovirus (JN819202) in the family *Picornaviridae* (Woo et al., 2012). As shown in Figure 1D, RCDaV polyproteins are divided into three different regions: region P1 encodes viral CPs, region P2 encodes protein 2AB and 2C, and region P3 encodes proteins 3A-3D known as viral protein-processing and genome-replication proteins. According to the conserved domain identified in the two polyproteins of RCDaV, at least six putative proteins were predicted, however, the boundaries and the precise cleavage sites of the structural and non-structural proteins need to be determined.

### *Cis*-Cleavage Activity of RCDaV 3Cpro

**FIGURE 2:**
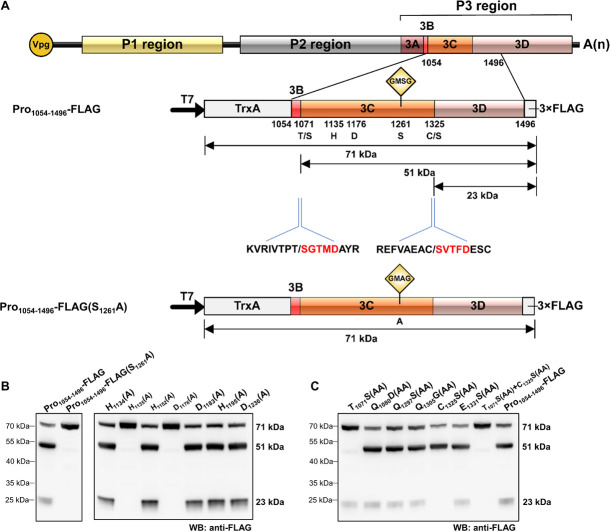
Analysis of rice curl dwarf-associated picornavirus (RCDaV) 3Cpro *cis*-cleavage activity. **(A)** Schematic diagrams of RCDaV genome organization, the 3 × FLAG tagged Pro_1054–1496_ fusion protein (Pro_1054–1496_-FLAG) and its mutant. Positions of the site-directed mutations are indicated. A substitution of S to A was introduced at the position 1261 in Pro_1054–1496_-FLAG to generate a mutant Pro_1054–1496_-FLAG(S_1261_A). The first five aa residues of the cleaved products were determined via the N-terminal Edman degradation sequencing, and are shown in red. Slashes indicate the cleavage sites. The calculated molecular masses of the cleaved products, including the vector-derived non-viral sequences, are indicated. **(B)** *In vitro cis*-cleavage activities of Pro_1054–1496_-FLAG and its mutants with a single aa residue substitution in the catalytic triad. **(C)** Identification of *cis*-cleavage sites of RCDaV 3Cpro by *in vitro cis*-cleavage assays using seven mutants, the dipeptide of predicted cleavage sites was mutated to AA.

The picornaviral genome is generally translated into large precursor polyproteins followed by proteolytic processing through self-released 3Cpro, which is a vital step for viral structural and non-structural proteins maturation. To determine the *cis*-cleavage activity of RCDaV 3Cpro, a gene segment containing 3Cpro domain (aa position 1,054–1,496 of polyprotein 2) was cloned into the modified pET32a vector to produce pET32a-Pro_1054–1496_-3 × FLAG (referred to as Pro_1054–1496_-FLAG thereafter) (Figure 2A). After 3 h expression in the *Escherichia coli* cell extract-based transcription/translation system (*E. coli* cell-free system), the expressed proteins were analyzed using western blot analysis with anti-FLAG antibody. The result showed that a 71 kDa protein band, the expected size of the intact Pro_1054–1496_-FLAG, as well as a 51 and a 23 kDa protein bands were detected (Figure 2B, lane 1). The presence of 71 kDa band indicated that the *cis*-cleavage was incomplete. We postulated that the 51 kDa protein band was the product autocatalytically cleaved from the N-terminus of 3Cpro and the 23 kDa protein band was cleaved from the C-terminus of 3Cpro. The intensity of the 23 kDa protein band was much weaker than that of the 51 kDa protein band, suggesting the *cis*-cleavage at 3Cpro N-terminus is more efficient than that at the C-terminus.

The catalytic triad of 3Cpro is a set of three coordinated amino acids in the active site of the enzyme. Each of these three key amino acids plays an essential role in the cleaving ability of the protease, and mutating these amino acids dramatically impacts 3Cpro cleavage activity (Dessens and Lomonossoff, 1991). To exclude the possibility that the protein bands described above were cleaved by bacterial-derived proteases and to identify the catalytic triad of RCDaV 3Cpro, aa residues involved in the proteolytic process were predicted through comparing the sequences and 3D structures of 3Cpros from RCDaV and hepatitis A virus (HAV) (Supplementary Figures 2A,B). Then the predicted catalytic triad was determined via site-directed mutagenesis analyses. The result showed that the cleavage bands of 51 kDa and 23 kDa vanished in the H_1135_(A), D_1176_(A) and S_1261_(A) mutants of Pro_1054–1496_-FLAG (Figure 2B). In classic picornaviral 3Cpro, the last amino acid of the catalytic triad is Cys (C), which is also the central amino acid of the core motif GxCG (Flint and Ryan, 1997). Interestingly, this Cys is replaced by Ser (S) in RCDaV 3Cpro, which results in a serine protease core motif GMSG (Figure 2B, lane 2).

To determine the exact *cis*-cleavage sites, we expressed Pro_1054–1496_-FLAG in *E. coli* BL21 (DE3) cells and purified it using FLAG beads. After being separated by SDS-PAGE, the 51 and 23 kDa products were subjected to N-terminal Edman sequencing, respectively. The results revealed that the five amino acids at the N-terminus of the 51 kDa product were SGTMD (aa position 1,072–1,076 of the polyprotein 2), suggesting the cleavage occurred at the T_1071_/S dipeptide (Figure 2A). The five amino acids at the N-terminus of the 23 kDa product were SVTFD (aa position 1,326–1,330 of the polyprotein 2), suggesting that the cleavage occurred at the C_1325_/S dipeptide (Figure 2A). To validate this result, we mutated the T_1071_S, C_1325_S dipeptide, or putative cleavage site QG, ES dipeptides to AA, and *cis*-cleavage analyses demonstrated that the 51 kDa product was not detected in the T_1071_S(AA) mutant and the 23 kDa product was not detected in the C_1325_S(AA) mutant (Figure 2C, lanes 1, 5). As expected, other mutants gave the same cleaved products as Pro_1054–1496_-FLAG (Figure 2C, lanes 2–4, 6, 8). In addition, the *cis*-cleavage activity of the double mutant [(T_1071_S(AA) + C_1325_S(AA)] was abolished (Figure 2C, lane 7). Taken together, these results indicate that the N- and C-terminal boundaries of the RCDaV 3Cpro are aa 1,072 and 1,325, and the cleavage occur at the T_1071_S and C_1325_S dipeptides.

### *Trans*-Cleavage Activity of RCDaV 3Cpro

**FIGURE 3:**
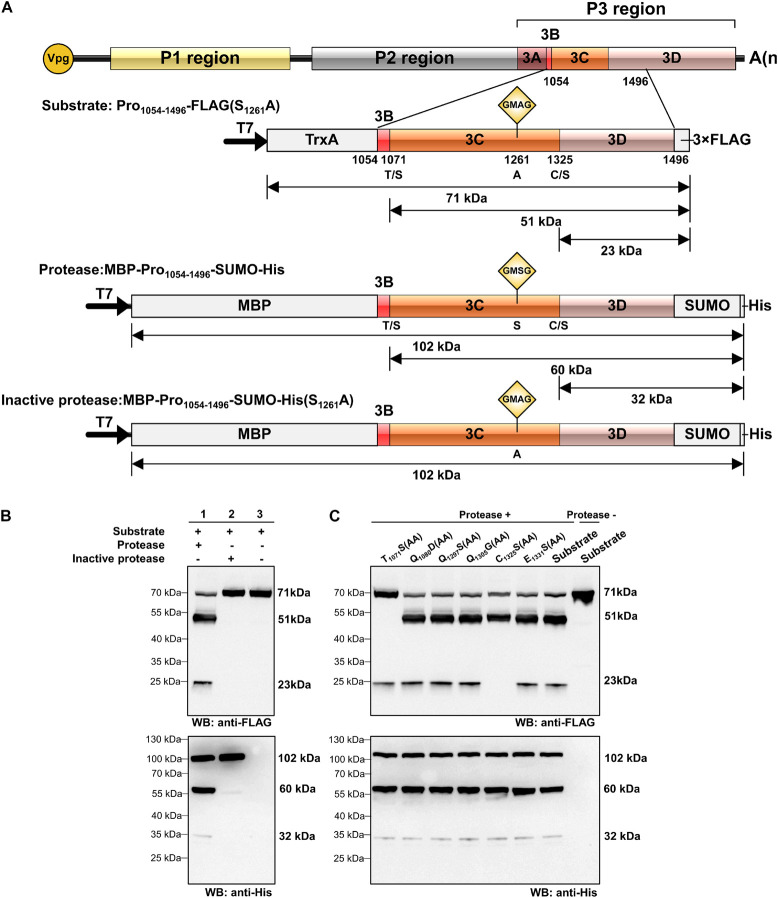
Analysis of rice curl dwarf-associated picornavirus (RCDaV) 3Cpro *trans*-cleavage activity. **(A)** Schematic diagrams of RCDaV genome, the 3 × FLAG tagged mutant Pro_1054–1496_-FLAG(S_1261_A), the MBP, SUMO and 6 × His tagged MBP-Pro_1054–1496_-SUMO-His and its mutant MBP-Pro_1054–1496_-SUMO-His (S_1261_A). The MBP-Pro_1054–1496_-SUMO-His was used as an active 3Cpro. The Pro_1054–1496_-FLAG(S_1261_A) and the MBP-Pro_1054–1496_-SUMO-His(S_1261_A) were used as the substrates and an inactive 3Cpro, respectively. **(B,C)** *In vitro trans*-cleavage activities of the MBP-Pro_1054–1496_-SUMO-His and its mutants. Pro_1054–1496_-FLAG(S_1261_A) or its mutants (used as the substrate) and MBP-Pro_1054–1496_-SUMO or MBP-Pro_1054–1496_-SUMO-His(S_1261_A) (used as the protease) were expressed individually for 1.5 h, mixed as indicated, and then incubated for another 1.5 h. The products were analyzed via western blot using an anti-FLAG or an anti-6 × His antibody. The predicted *trans*-cleavage sites in the substrate of RCDaV 3Cpro were mutated to AA.

Using the preliminary experiments, we determined that the optimum temperature for protein expression was 30°C and the optimum temperature for cleavage was 28°C. In order to optimize the cleavage efficiency, the reaction temperature was set at 30°C for the first 1.5 h and 28°C for the last 1.5 h in the *trans*-cleavage experiment. To test the *trans*-cleavage activity of RCDaV 3Cpro, pET28a-MBP-Pro_1054–1496_-SUMO-His (MBP-Pro_1054–1496_-SUMO-His) and pET28a-MBP-Pro_1054–1496_-SUMO-His(S_1261_A) were constructed and expressed in the *E. coli* cell-free system. The expressed MBP-Pro_1054–1496_-SUMO-His and MBP-Pro_1054–1496_-SUMO-His(S_1261_A) were used as the functional and the non-functional proteases, respectively, and the *cis*-cleavage-defective mutant Pro_1054–1496_-FLAG(S_1261_A) was used as the substrate (Figure 3A). Western blot analyses showed that MBP-Pro_1054–1496_-SUMO-His (protease) efficiently cleaved Pro_1054–1496_-FLAG(S_1261_A) (substrate) to produce 51 kDa and 23 kDa cleavage products (Figure 3B, lane 1), while the substrate incubated alone remained intact (Figure 3B, lane 3). As expected, the substrate incubated with MBP-Pro_1054–1496_-SUMO-His(S_1261_A) (inactive Protease) did not yield the cleaved protein bands (Figure 3B, lane 2), indicating that RCDaV 3Cpro is enzymatically active and able to mediate *trans*-cleavage.

To compare the *trans-* and *cis-* cleavage sites, we introduced the mutations into the substrate and then incubated them individually with MBP-Pro_1054–1496_-SUMO-His. Western blot analyses showed that the *trans*-cleavage sites also occurred at the T_1071_/S and C_1325_/S dipeptides (Figure 3C). To optimize the *trans*-cleavage efficiency, we also analyzed the enzymatic activity of tag-free RCDaV 3Cpro_1072–1325_ (Supplementary Figure 3A). The result showed the tag-free 3Cpro_1072–1325_ could also catalyze the cleavage of the substrates used in this study (Supplementary Figure 3B, lane 1). Since the *trans*-cleavage activity of tag-free 3Cpro_1072–1325_ was relatively higher than MBP-Pro_1054–1496_-SUMO-His (Supplementary Figure 3B), we used the tag-free 3Cpro_1072–1325_ in the subsequent assays. Based on these results, RCDaV 3Cpro is identified as a serine protease with both *cis*-cleavage and *trans*-cleavage activity.

### Precisely Mapping the Cleavage Sites on RCDaV Polyproteins

**FIGURE 4:**
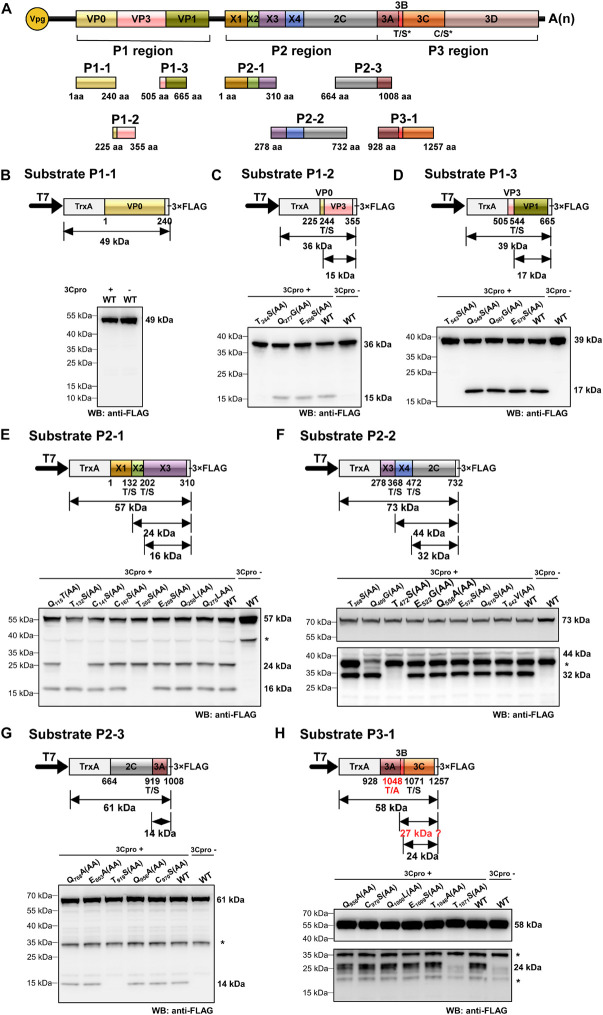
Identification of cleavage sites in rice curl dwarf-associated picornavirus (RCDaV) polyproteins using *in vitro trans*-cleavage assay. **(A)** Schematic representation of protein segments from the three regions of RCDaV. **(B–D)** *In vitro trans*-cleavage analyses using the P1-1, P1-2, and P1-3 protein segments from P1 region as the substrates. **(E–G)** *In vitro trans*-cleavage analyses using the P2-1, P2-2, and P2-3 protein segments from P2 region as the substrates. **(H)** *In vitro trans*-cleavage analysis using the P3-1 protein segment from P3 region as the substrate. Site-directed mutants of the predicted cleavage sites were also used in individual assays. The calculated molecular mass of each protein segment is shown on the right side of the figure. The asterisks in **(E–H)** indicate non-specific protein bands. The lower panel blots in **(F,H)** were exposed for longer time to show the bands. Predicted T/A cleavage site is shown with a dashed line in **(H)**, since processing was not detected at this site in the *in vitro* assays. Numbers below the boxes indicate amino acids positions.

Polyproteins of picornaviruses are processed co- and post-translationally into 10–12 mature proteins (Lange et al., 2014). To determine the precise boundaries of functional viral proteins proteolytically cleaved by RCDaV 3Cpro, we analyzed the cleavage sites in RCDaV polyproteins via the *trans*-cleavage assays. We selected gene segments containing the boundaries of the predicted viral proteins to generate their 3 × FLAG-tagged fusion proteins as the substrates and used the tag-free 3Cpro_1072–1325_ as the protease (Figure 4A). After confirming the *trans*-cleavage of the substrates, mutations (e.g., QG-AA, ES-AA, TS-AA, and CS-AA) were introduced individually into the substrates through site-directed mutagenesis (Figures 4B–H and Supplementary Table 2). The cleavage sites were then determined by comparing the cleaved mutant substrates with the wild type substrates.

Some picornaviruses possess leader protein preceding the P1 region, such as the genera *Aphthovirus* and *Cardiovirus* in the family *Picornaviridae* (Devaney et al., 1988; Chen et al., 1995). The leader proteins of aphthoviruses are identified as papain-like cysteine proteinases that are able to self-cleave carboxy terminally (Devaney et al., 1988). In *trans*-cleavage assays, the substrate P1-1 (aa position 1–240 of the P1 region) produced a single 49 kDa protein band, in the presence or absence of 3Cpro_1072–1325_, indicating that no *trans*-cleavage catalyzed by RCDaV 3Cpro or *cis*-cleavage catalyzed by L protein occurred (Figure 4B). Using substrate P1-2 (aa position 225–355 of the P1 region), we found that besides the intact 36 kDa protein band, an additional 15 kDa cleavage protein band catalyzed by 3Cpro_1072–1325_ was observed, except the T_244_S(AA) mutant resulted in a complete inhibition of cleavage processing (Figure 4C). Besides the 39 kDa substrate P1-3 (aa position 505–665 of the P1 region), a 17 kDa cleavage protein catalyzed by 3Cpro_1072–1325_ was produced except the T_543_S(AA) mutant (Figure 4D), suggesting that the T_543_S dipeptide in P1-3 is important for the recognition by 3Cpro_1072–1325_. Analyses of the P1 region revealed the presence of two cleavage sites (T_244_/S and T_543_/S), thus the P1region can be processed in three CPs, but L protein is absent.

**FIGURE 5:**
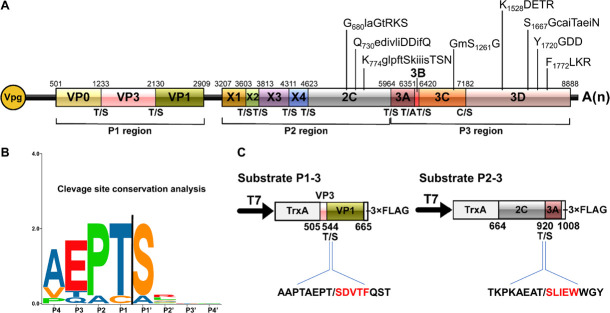
Conservation analysis of cleavage sites in rice curl dwarf-associated picornavirus (RCDaV) polyproteins. **(A)** Schematic representation of the elaborate genome organization. The genome maps and each gene boxes were drawn to scale. Nucleotide position of individual protein is indicated above each gene box. The structural proteins are named as reported previously (Roberts and Groppelli, 2009). The cleavage sites are indicated at the borders between the two cleaved proteins. The conserved picornaviral motifs are indicated above the diagram. **(B)** Sequence logos of RCDaV cleavage sites. The P4–P1/the P1′–P4′ aa of cleavage sites are shown. Amino acids are color-coded according to their physicochemical characteristics. Neutral and polar, green; basic, blue; acidic, red; neutral and hydrophobic, black. Amino acids are shown as one-letter standard code. **(C)** The first five amino acids (red) in cleavage products of substrates P1-3 and P2-3 were determined through N-terminal Edman degradation sequencing. Slashes indicate the cleavage sites.

Further analyses of the RCDaV P2 region using *trans*-cleavage assays showed that this region can be cleaved into five proteins (Figures 4E–G). The *trans*-cleavage occurred at five highly conserved T/S dipeptides (aa position 132–133, 202–203, 368–369, 472–473, and 919–920, respectively), but not at the C/S or the Q(E)/G(S) dipeptides (Figures 4E–G). The cleavage at the T/S dipeptides in P2 region produce five proteins: a 15 kDa protein (referred to as X1), an 8 kDa protein (X2), a 20 kDa protein (X3), a 12 kDa protein (X4), and a 50 kDa 2C protein. We tentatively named the first four proteins as X1–X4 because they do not possess any motifs known in the classic picornaviral 2A or 2B proteins (Tseng and Tsai, 2007; Boros et al., 2014), and have no sequence similarities to those identified in other picornaviruses. In addition, unexpected protein bands, indicated with asterisks, were found in the absence of RCDaV 3Cpro (Figures 4E–H and Supplementary Figures 4A,B). We postulate that these protein bands might be the products of endogenous proteases in the cell-free system. The 2C protein was predicted as a helicase, one of the most conserved proteins encoded by viruses in the order *Picornavirales*. Picornaviral 2C proteins are all superfamily III helicases and contain three conserved motifs: GxxGxGK(S/T) (motif A), WWWxxDD (motif B), and KGx_4_Sx_5_(S/T)(S/T)N (motif C) (Hales et al., 2008). The motif A of RCDaV 2C is G_680_LAGTRKS and its third “G” is substituted by “R” at the aa position 685. The motifs B and C are DIVLID_737_D and K_774_GLPFTSKIIISTSN, respectively (Figure 5A).

In the P3 region, since the cleavage sites of 3B/3Cpro and 3Cpro/3D have already been determined (Figures 2C, 3C), the only one that remained to be analyzed was at the 3A/3B junction. Results showed that the approximate 27 kDa protein band was absent in the cell-free system, indicating that the cleavage of 3A/3B is difficult to be detected in our *in vitro* assay (Figure 4H and Supplementary Figure 4B). According to the conserved cleavage sites concluded from above experiments, we speculate that the PEPT_1048_/A sequence might be the cleavage site between 3A and 3B. This prediction is consistent with the rule that the fourth residue of VPg N-terminus is a tyrosine (Y) for linkage to the 5′ end of RCDaV genome (Rothberg et al., 1978). The cleavage at the T_1048_/A dipeptide can produce a 15 kDa 3A and a 2.6 kDa 3B proteins. Consequently, we conclude that RCDaV polyproteins can produce 12 mature proteins in the order: P1 region (VP0, VP3, VP1); P2 region (X1, X2, X3, 2B, 2C); P3 region (3A, 3B, 3C, and 3D) (Figure 5A).

The above results of 3Cpro encouraged us to further characterize the cleavage site pattern of RCDaV. Except C/S dipeptide at the 3C/3D junction and T/A dipeptide at the 3A/3B junction, the RCDaV 3Cpro-mediated cleavage occurs at highly conserved T/S dipeptides. We performed cleavage site conservation analysis, and the result shows that the T/S dipeptide at P1/P1′ position is highly conserved (Figure 5B), which differs from the classic Q(E)/G(S) cleavage sites identified in most picornaviruses. In addition, several aa residues preceding the T/S cleavage site are relatively conserved, i.e., A(V)EP (Figure 5B). To validate this finding, the cleaved products from substrate P1-3 and P2-3 were purified and subjected to N-terminal Edman degradation sequencing. The results agreed with the finding described above, indicating the results of *trans*-cleavage assays are reliable (Figure 5C).

### Phylogenetic Relationships Between RCDaV and Other Viruses in the Order *Picornavirales*

**FIGURE 6:**
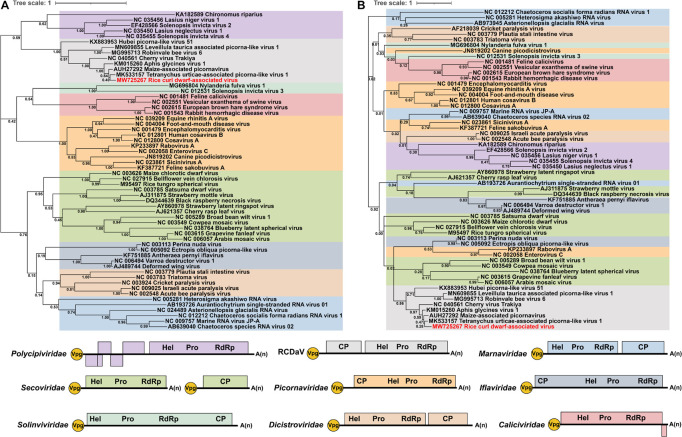
Phylogenetic analyses using the RdRP and 3Cpro amino acid sequences from 58 viruses in the order *Picornavirales*. The phylogenic trees of RdRP **(A)** and 3Cpro **(B)** amino acid sequences were constructed using the maximum likelihood method with 1000 bootstraps. Among these 58 viruses, 10 were from *Picornaviridae*, four from *Caliciviridae*, two from *Solinviviridae*, five from *Polycipiviridae*, six from *Marnaviridae*, five from *Dicistroviridae*, five from *Iflaviridae*, 13 from *Secoviridae*, seven from unclassified picornaviruses, and one from rice curl dwarf-associated picornavirus (RCDaV). Viruses from the same family are shown in the same color. The name of RCDaV is shown in red. The bottom panel shows the schematic diagram of genome organizations of different families used in this analysis. Virus names and their GenBank or Refseq accession numbers are listed in Supplementary Table 3.

In addition to RCDaV, we also retrieved RdRP or 3Cpro aa sequences of 50 representative species from eight officially classified families and seven unclassified picornaviruses in the order *Picornavirales* from the NCBI database (Supplementary Table 3). Previous studies have reported that these unclassified bicistronic picornaviruses discovered from arthropods and plants that cluster into a highly divergent clade (François et al., 2019; Koloniuk et al., 2020; Yasmin et al., 2020), may represent a novel family in the order *Picornavirales* (Yasmin et al., 2020). Phylogenetic analysis indicated that RCDaV RdRP clustered together with RdRPs from these seven picornaviruses, and formed a clade distinct from eight officially classified families (Figure 6A). Moreover, the RdRP aa sequence identities between RCDaV and seven unclassified picornaviruses were 44.3–87.9%, while between RCDaV and other officially classified picornaviruses, the RdRP aa sequence identities were less than 30% (Supplementary Tables 5A,B). These results indicate that RCaDV should be grouped in this distinct clade of unclassified viruses in the order *Picornavirales*.

At the same time, the previous study observed the core motif GxSG of 3Cpros in this unclassified viruses group differs from that of other picornaviruses (Yasmin et al., 2020). In our studies, sequence alignment results also show that RCDaV and these seven picornaviruses share the similar core motif GxSG (Supplementary Figure 5), suggesting that their 3Cpros are typical serine protease and different from the chymotrypsin-like cysteine protease of other picornaviruses, except 3Cpros of Heterosigma akashiwo RNA virus in the family *Marnaviridae*, blueberry latent spherical virus in the family *Secoviridae* and the viruses in the family *Polycipiviridae* (Olendraite et al., 2017; Mann and Sanfaçon, 2019). The phylogenetic tree based on 3Cpro aa sequences also indicates that RCDaV is phylogenetically related to the seven unclassified picornaviruses. However, 3Cpros of these eight viruses show a distant evolutionary relationship with the serine proteases of the family *Marnaviridae*, *Secoviridae*, and *Polycipiviridae* (Figure 6B).

### This Distinct Clade of Picornaviruses Has the Conserved EPT/S Cleavage Site Pattern

**FIGURE 7:**
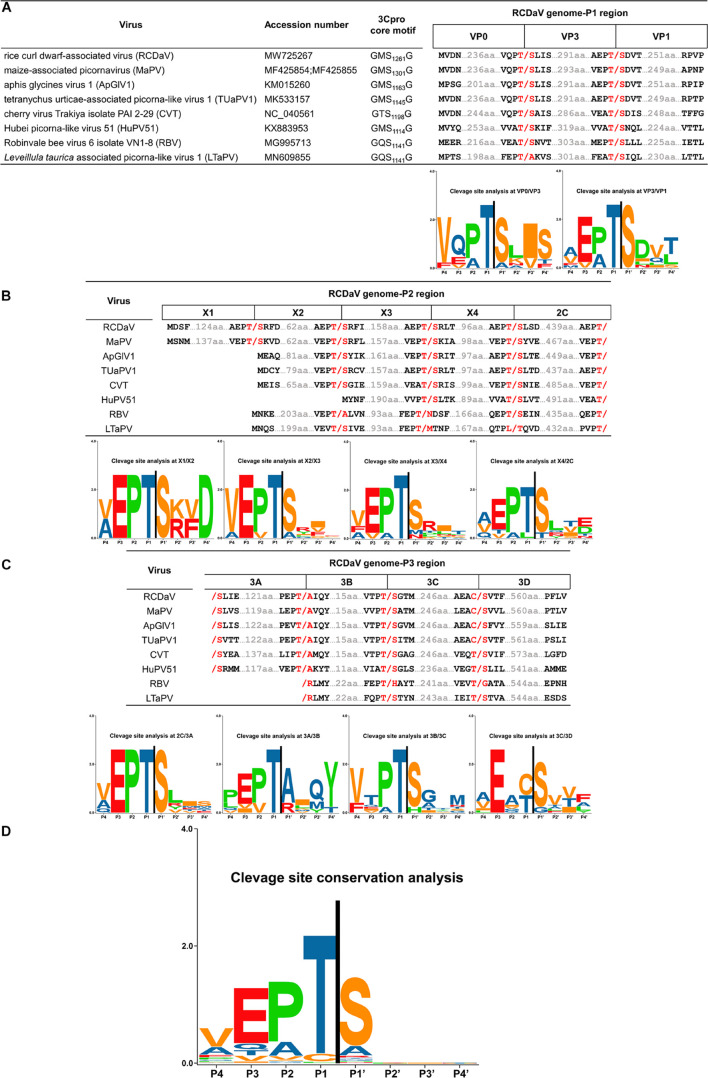
Predicted cleavage sites in polyproteins of the seven unclassified picornaviruses based on rice curl dwarf-associated picornavirus (RCDaV) cleavage sites. **(A–C)**, Predicted cleavage sites in P1, P2, and P3 regions are shown. The GxSG motif of each virus and conservation analysis of each cleavage sites are also shown. The viral proteins were named according to the cleavage sites, the positions, and the sequence lengths of proteins. Compared with the other viruses, Robinvale bee virus 6 isolate VN1-8 (RBV) and *Leveillula taurica* associated picorna-like virus 1 (LtaPV) lack the 3A protein. **(D)** Conservation analysis of all the predicted cleavage sites in the eight picornaviruses. Amino acids are color-coded according to their physicochemical characteristics. Neutral and polar, green; basic, blue; acidic, red; neutral and hydrophobic, black. All amino acids are shown as one-letter standard code.

Previous researchers have noticed the unusual taxonomic status of several unclassified picornaviruses and the core GxSG motif of their 3Cpros; however, cleavage sites proteolytically processed by their 3Cpros have not been determined precisely (Yasmin et al., 2020). To find out if the highly conserved T/S dipeptide is also present in these viruses, we compared the sequences of the polyproteins of seven unclassified viruses at putative cleavage sites using cleavage positions of RCDaV polyproteins as the reference (Figure 7). As expected, the T/S dipeptides at the corresponding positions were highly conserved, and the aa residues flanking these cleavage sites were slightly different (Figure 7). We performed cleavage site conservation analysis for each junction. At some junctions, such as 3C/3D, the cleavage site contains C or T at the P1 position (Figure 7C), and in most cases, the 3A/3B junction contains A at the P1′ position. Highly conserved EP exists at most P2 and P3 positions, with occasionally EA, EV, or VP (Figures 7A–C). The less conserved residues at the P4 position are V, A, or L (Figures 7A–C). Through alignment analysis, we found very low aa residue similarities at the P2′–P4′ positions at different junctions of each virus (Figure 7). However, aa residues at some junctions in different viruses are similar, especially at the junctions between CPs, such as the consensus VQPT/SLIS sequence at the VP3/VP0 junction of RCDaV, MaPV, ApGlV1, and TUaPV1 (Figure 7A). Also the consensus sequence at their VP0/VP1 junction is A(V)EPT/SDVT (Figure 7A). Nevertheless, aa residues of CVT, Hubei picorna-like virus 51 (HuPV51), Robinvale bee virus 6 isolate VN1-8 (RBV) and *Leveillula taurica* associated picorna-like virus 1 (LtaPV) are more variable at the P2′–P4′position, probably due to the more distant homology relationships (Figure 7). The conservation analysis for all the sites reveals that the EPT/S sequence is the conserved cleavage site of these eight viruses (Figure 7D).

**FIGURE 8:**
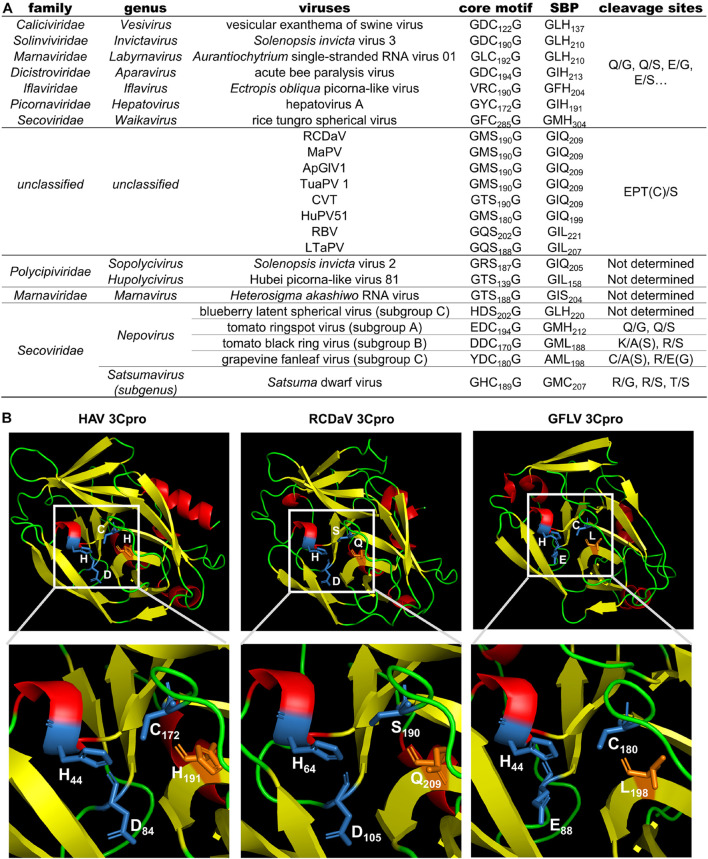
Comparison analyses of the 3Cpro sequence and 3D structure from viruses in the order *Picornavirales.* **(A)** Comparison results of the core motif, partial substrate binding pocket (SBP) and cleavage sites of 3Cpros from viruses in the order *Picornavirales.* The cleavage sites of viruses from eight official families were obtained or predicted from previous studies (Allaire et al., 1994; Hemmer et al., 1995; Blom et al., 1996; Thole and Hull, 1998; Ye et al., 2012; Kristensen et al., 2018; Mann and Sanfaçon, 2019; Sanfaçon et al., 2020). **(B)** The 3D structure comparison of 3Cpros from HAV, RCDaV and GFLV. The 3D structure of HAV 3Cpro was downloaded from the RCSB PDB database (https://www.rcsb.org/, ID: 1QA7). The 3D structures of 3Cpros from RCDaV and GFLV were predicted using the HAV 3Cpro as the template through SWISS-MODEL homology-modeling server. The secondary structures are shown in different colors: red, helix; yellow, sheet; green, loop. The residues of catalytic triad are highlighted by blue and the key residues of S1 position of the SBP are highlighted in orange.

Previous studies reported the conserved His in the S1 position of the substrate binding pocket (SBP) of most chymotrypsin-like viral proteases has a connection with the recognition of cleavage sites with Gln (or Glu) at the P1 position (Mann and Sanfaçon, 2019). While the cleavage specificity of 3Cpros encoded by some nepoviruses (e.g., grapevine fanleaf virus, GFLV) may be different due to the replacement of His with Leu in the S1 position of the SBP (Margis and Pinck, 1992). We also analyzed the 3Cpro aa sequences of the eight viruses and found that the conserved His changes to Gln or Leu in the S1 position of the SBP, which could be one of the reasons why 3Cpro of the eight viruses recognize different cleavage sites (Figures 8A,B).

**FIGURE 9:**
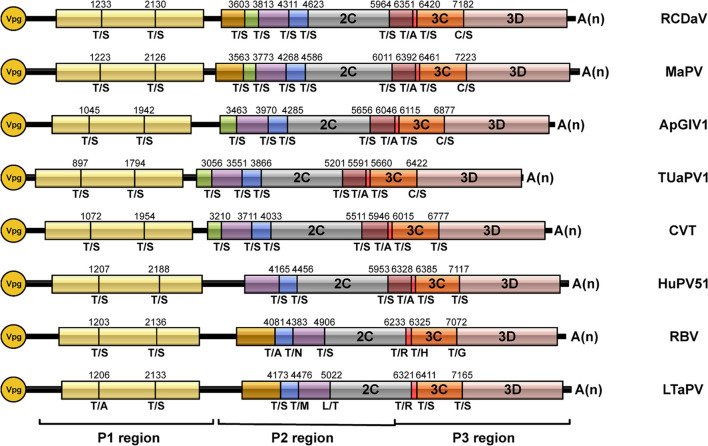
Genome organizations of rice curl dwarf-associated picornavirus (RCDaV) and seven unclassified picornaviruses. The predicted cleavage sites are shown.

**FIGURE 10:**
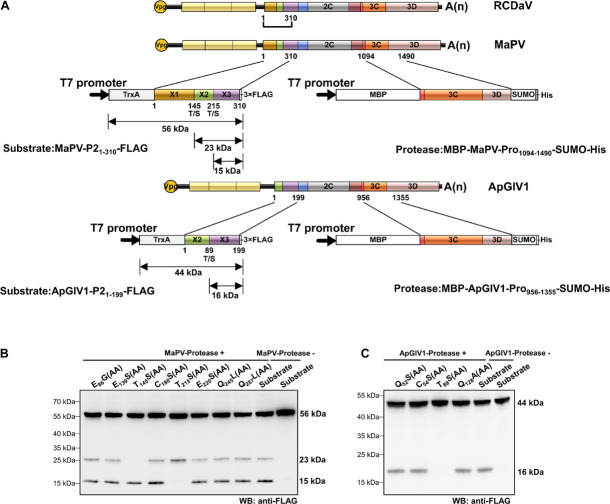
Analyses of cleavage sites in the N-terminus of P2 regions of MaPV and ApGlV1 using *in vitro trans*-cleavage assays. **(A)** Schematic representation of the expressed substrates and the expressed proteases. MaPV-P2_1–310_ and ApGlV1_1–199_ were used as the substrates, and MBP-MaPV-Pro_1094–1490_-SUMO-His and MBP-ApGlV1-Pro_956–1355_-SUMO-His were used as the proteases. The calculated molecular mass of individual cleaved products is indicated. The predicted cleavage sites are shown as described in Figure 9. **(B)** *In vitro trans*-cleavage analysis of the N-terminus of P2 regions of MaPV and their mutants. The indicated amino acids in the mutants were mutated to AA. **(C)** *In vitro trans*-cleavage analysis of the N-terminus of P2 regions of ApGlV1 and their mutants. The indicated amino acids in the mutants were mutated to AA.

According to the predicted cleavage sites, we illustrated the precise genome organizations of these viruses (Figure 9). To verify these predictions for T/S cleavage sites, we analyzed two viruses (MaPV and ApGlV1). The substrate MaPV-P2_1–310_ contains the 1–310 aa residues of MaPV P2 region, and the substrate ApGlV1-P2_1–199_ contains the 1–199 aa residues of ApGlV1 P2 region. These two substrate genes were synthesized, and inserted in the modified pET32a vector, respectively (Figure 10A). The *trans*-cleavage assays using the cell-free system as described above showed that the 56 kDa MaPV-P2_1–310_ protein was cleaved by MaPV 3Cpro (MBP-MaPV-Pro_1094–1490_-SUMO-His) to produce 23 and 15 kDa proteins (Figure 10B). T_145_S(AA) mutant abolished the production of the 23kDa protein, while T_215_S(AA) mutant abolished the production of the 15 kDa protein (Figure 10B), suggesting that EPT_145_/S and EPT_215_/S are the cleavage sites of MaPV 3Cpro. Mutations of other predicted sites had no effect on the cleavage activity of MaPV 3Cpro (Figure 10B). As expected, the ApGlV1-P2_1–199_ substrate and its Q_52_S(AA), C_54_S(AA), and Q_128_A(AA) mutants were cleaved by ApGlV1 3Cpro (MBP-ApGlV1-Pro_956–1355_-SUMO-His) to produce a 16 kDa protein band, while the T_89_S(AA) mutant was not cleaved (Figure 10C), suggesting that EPT_89_/S is the site cleaved by ApGlV1 3Cpro. These results confirm that the EPT/S sequence is the conserved cleavage site recognized by 3Cpros from viruses in this group.

## Discussion

Proteases are classified in clans and families, based on their catalytic types, phylogeny, and molecular structures (Rawlings et al., 2018; Mann and Sanfaçon, 2019). A recent study indicated that the plant positive-stranded RNA virus-encoded proteases can be grouped into two clans: chymotrypsin-like cysteine or serine proteases clan (clan PA) and the papain-like cysteine proteases clan (clan CA) (Mann and Sanfaçon, 2019). The structure of the cellular chymotrypsin is characterized by a double-barrel fold, which is shared by the viral chymotrypsin-like proteases (Mann and Sanfaçon, 2019). The activity of chymotrypsin depends on the catalytic triad containing His, Asp/Glu, and nucleophile Ser, which are brought together in the 3D structure (Dessens and Lomonossoff, 1991; Sárkány and Polgár, 2003). The Ser is conserved in the viral serine proteases, however, in picornaviral 3Cpros, the nucleophile Ser is replaced by Cys to form the classic core motif GxCG (Allaire et al., 1994). It has been shown that this core motif is essential for the 3Cpro activity (Dessens and Lomonossoff, 1991; Blair et al., 1996; Thole and Hull, 1998). In this study, we identified a novel rice-associated picornavirus, RCDaV, which encodes a serine 3Cpro with the catalytic triad composed of His_1135_, Asp_1176_ and Ser_1261_ (Figure 2B). And the mutation of GMS_1261_G to GMA_1261_G abolished the *cis*- and *trans*-cleavage activities of RCDaV 3Cpro (Figure 2B, 3B, left panel). Meanwhile, the substitution of His_1135_ and Asp_1176_ with Aln also abolished the proteolytic activity of RCDaV 3Cpro (Figure 2B). Based on these findings, RCDaV 3Cpro is a chymotrypsin-like serine protease which is different from the chymotrypsin-like cysteine proteases of other picornaviruses, except 3Cpros of Heterosigma akashiwo RNA virus in the family *Marnaviridae*, blueberry latent spherical virus in the family *Secoviridae* and the viruses in the family *Polycipiviridae* (Figure 8A; Olendraite et al., 2017; Mann and Sanfaçon, 2019).

Cleavage sites of most picornaviral 3Cpros are known as Q(E)/G(S) dipeptides (Seipelt et al., 1999; Sanfaçon, 2015). However, using *in vitro* cleavage assays, the conserved EPT/S cleavage sites recognized by RCDaV 3Cpro were identified (Figure 4), and the viral polyproteins can be processed into 12 mature proteins via *cis*- or *trans*-cleavage manner (Figures 2–4). Up to now, very few cleavage site proteolytically processed by serine protease in picornaviruses has been experimentally verified (Figure 8A). Additionally, one T/S cleavage site has been identified in satsuma dwarf virus (SDV) catalyzed by its cysteine protease previously (Iwanami et al., 1998). However, the preceding aa sequence of T/S cleavage site from SDV is AQ which has no similarity with that of RCDaV (EP), and the existence of other R/G(S) cleavage sites indicates that the T/S cleavage site is not conserved in SDV (Iwanami et al., 1998). Therefore, compared with the previous identified 3Cpro cleavage sites, the highly conserved cleavage site EPT/S of RCDaV 3Cpro is relatively uncommon in picornaviruses.

Allaire et al. (1994) have shown that the specific recognition of Gln (Q) or Glu (E) at the P1 position is conferred by the conserved His in the S1 position of the SBP of most chymotrypsin-like viral 3Cpros. Although some 3Cpros from viruses in the family *Secoviridae* substitute Leu (nepoviruses of subgroup A and B, sequiviruses) or Cys (satsumavirus) for the conserved His in their SBPs (Mann and Sanfaçon, 2019; Figure 8A), and this may lead some nepovirus proteases recognize a variety of different cleavage sites with Asn, Asp, Arg, Lys, Cys, or Gly at the P1 position, and sequivirus proteases recognize Asp, Ser (Sanfaçon et al., 2009), while satsumavirus proteases recognize Thr or Arg (Sanfaçon et al., 2020). In our study, the conserved His in the S1 position of the SBP of RCDaV 3Cpro is replaced by Gln (Figure 8A). The comparison analysis of 3D structures of 3Cpros from HAV, RCDaV, and GFLV shows that the Gln occupies the position of the His in RCDaV 3Cpro (Figure 8B). Moreover, sequence alignment analyses reveal that the conserved His in this position of MaPV, ApGlV1, TUaPV1, CVT, and HuPV51 is also replaced by Gln (Figure 8A). To our surprise, this position of 3Cpros from RBV and LtaPV is replaced by Leu, which is similar with some viruses in the family *Secoviridae* mentioned above. However, 3Cpros from RBV and LtaPV still recognize the EPT/S cleavage sites, which differ from the 3Cpros with Leu in their S1 position of the SBP of some secoviruses. Thus, the reason underlying the recognized Thr or Cys in the P1 position may be complex and remains to be further explored.

In recent years, a great number of new viruses were discovered through RNA-seq technology. Among these new viruses, picornaviruses account for a considerable proportion. Yasmin et al. (2020) identified a novel picorna-like virus ApGlV1 through high-throughput sequencing. They have found that ApGlV1 differs from viruses in the officially classified families of picornaviruses, and is phylogenetically closely related to a clade of unclassified viruses with similar characteristics. Therefore, they considered these viruses might represent a new family under the order *Picornavirales.* Yasmin et al. (2020) also noticed that the core motif of 3Cpros from these viruses have changed to the GxSG type. However, their predicted cleavage sites on the polyproteins of ApGlV1 were not mapped correctly based on our results (Figure 7), probably because they did not realize that the cleavage specificity of this type of 3Cpros might be different from the classic picornaviral 3Cpros. Thus, how the polyproteins of these unclassified viruses are processed into mature proteins is still unknown, which limits the further investigation of them. In our study, we prove that RCDaV clusters together with ApGlV1 into this distinct clade (Figure 6A), and all the viruses in this clade encode the similar serine proteases (Figure 6B). More importantly, we precisely mapped the EPT/S cleavage sites on the polyproteins of these picornaviruses (Figures 7A–C), suggesting the cleavage specificity of this type of serine protease is highly conserved. Based on these EPT/S cleavage sites, we illustrated the precise genome organizations of these viruses (Figure 9), and several predicted cleavage sites of MaPV and ApGlV1 were also verified experimentally (Figures 10A,B). These results indicate that EPT/S is the conserved cleavage site on the polyproteins from picornaviruses in this clade. The results in this study further support the previous view that this clade of picornaviruses may represent a new family, and we move forward demonstrating that their 3C serine proteases and their cleavage sites might be an essential characteristic of this clade of picornaviruses.

In summary, we have identified a novel picornavirus, rice curl dwarf-associated virus (RCDaV). Amino acid sequence alignment of RdRPs showed that RCDaV and seven picornaviruses share relatively high sequence identities ranging from 44.3 to 87.9% (Supplementary Table 5B). RCDaV and seven unclassified picornaviruses cluster into an independent clade which is distinct from eight officially classified families in the order *Picornavirales*. These viruses share similar genome organizations and encode the similar functional 3Cpros which are chymotrypsin-like serine proteases recognizing the conserved EPT/S cleavage sites. However, the molecular mechanism of how these 3Cpros recognize and cleave this uncommon EPT/S sites remains unclear. The impact of RCDaV on rice production, and its genomic function as well as its transmission mode in fields require further studies.

## Data Availability Statement

The datasets presented in this study can be found in online repositories. The names of the repository/repositories and accession number(s) can be found below: https://www.ncbi.nlm.nih.gov/genbank/, MW725267.

## Author Contributions

TZ and CL: writing—original draft preparation. DW: methodology. MC: validation. QW, YX, and SG: software. SF: formal analysis. JW and XZ: writing—review and editing. JW: funding acquisition. All authors have read and agreed to the published version of the manuscript.

## Conflict of Interest

The authors declare that the research was conducted in the absence of any commercial or financial relationships that could be construed as a potential conflict of interest.

## Publisher’s Note

All claims expressed in this article are solely those of the authors and do not necessarily represent those of their affiliated organizations, or those of the publisher, the editors and the reviewers. Any product that may be evaluated in this article, or claim that may be made by its manufacturer, is not guaranteed or endorsed by the publisher.

## Footnotes

1
https://talk.ictvonline.org/ictv-reports/ictv_9th_report/positive-sense-rna-viruses-2011/w/posrna_viruses/227/picornavirales

2
http://www.ictvonline.org/

3
www.picornaviridae.com

4
http://rice.plantbiology.msu.edu/

5
https://www.ncbi.nlm.nih.gov/Structure/cdd/wrpsb.cgi

6
https://itol.embl.de

## Supplementary Material

Supplementary Figure 1 Rice curl dwarf-associated picornavirus (RCDaV) was detected by RT-PCR in symptomatic rice and barnyard grass plants using RCDaV specific primers.

Supplementary Figure 2 Protein 3D structure prediction of rice curl dwarf-associated picornavirus (RCDaV) 3Cpro. **(A)** Using the structure of HAV 3Cpro as the template, the 3D structure of RCDaV 3Cpro was predicted through SWISS-MODEL homology-modeling server. The color on the sequence indicates the model quality, orange, low quality; blue, high quality. The mutated amino acid sites in *cis*-cleavage assays to verify catalytic triad of RCDaV 3Cpro are indicated by arrows. The red arrows represent the catalytic triad of RCDaV 3Cpro, while greens ones represent non-effective sites. **(B)** The comparison of the 3D structure of 3Cpros from RCDaV and HAV. The secondary structures are shown in different colors: red, helix; yellow, sheet; green, loop. The residues of catalytic triad are highlighted by blue.

Supplementary Figure 3 *Trans*-cleavage activities of tag-free rice curl dwarf-associated picornavirus (RCDaV) 3Cpro_1072–1325_. **(A)** Schematic representation of tag-free RCDaV 3Cpro_1072–1325_. **(B)** Substrate Pro_1054–1496_-FLAG(S_1261_A) was incubated with MBP-Pro_1054–1496_-SUMO-His or tag-free RCDaV 3Cpro_1072–1325_ in the *trans*-cleavage assay followed by western blot analysis.

Supplementary Figure 4 The original images of Figures 4F **(A)** and 4H **(B)**.

Supplementary Figure 5 Sequence alignment of 3Cpros from rice curl dwarf-associated picornavirus (RCDaV) and the seven unclassified picornaviruses. The core motifs of these 3Cpros are highlighted by red box.

Supplementary Table 1 All primers used in this study.

Supplementary Table 2 Details of the expressed genes and their vectors.

Supplementary Table 3 Viruses used in the phylogenetic analyses.

Supplementary Table 4 The BLASTp search using the RNA_dep_RNAP domain of rice curl dwarf-associated picornavirus (RCDaV).

Supplementary Table 5 Pairwise sequence comparison of rice curl dwarf-associated picornavirus (RCDaV) with other viruses in the order *Picornavirales*. **(A)** Pairwise amino acid sequence identities (%) of the P1, P2, P3, 3C, and 3D proteins encoded by RCDaV compared to the representative members in the eight official families. **(B)** Pairwise nucleotide and amino acid sequences identities (%) of RCDaV compared with the seven unclassified picornaviruses. %N, nucleotide sequence identities (%); %A, amino acid sequence identities (%).
