# Supplementary material for: A Novel Rice Curl Dwarf-Associated Picornavirus Encodes a 3C Serine Protease Recognizing Uncommon EPT/S Cleavage Sites
Source: Front Microbiol. 2021 Oct 13;12:757451. doi: 10.3389/fmicb.2021.757451 (PMC8549817; doi:10.3389/fmicb.2021.757451)
Supplement: Supplementary Figure 1 — Rice curl dwarf-associated picornavirus (RCDaV) was detected by RT-PCR in symptomatic rice and barnyard grass plants using RCDaV specific primers. [file Data_Sheet_1.zip › Table S4.DOCX]

**Table S4. The BLASTp search using the RNA_dep_RNAP domain of RCDaV.**

| **description** | **viruses** | **family** | **genus** | **GenBank accession no.** | **Query Cover** | **Per.Ident** |
| --- | --- | --- | --- | --- | --- | --- |
| polyprotein | *Maize-associated picornavirus* | unclassified | unclassified | AUH27292.1 | 99% | 90.6% |
| polyprotein | *Tetranychus urticae-associated picorna-like virus 1* | unclassified | unclassified | QIN54759.1 | 99% | 89.9% |
| non-structural polyprotein | *Aphis glycines virus 1* | unclassified | unclassified | AHC72013.1 | 100% | 83.2% |
| replicase | *Cherry virus Trakiya* | unclassified | unclassified | YP_009551963.1 | 99% | 67.2% |
