# Supplementary material for: A Novel Rice Curl Dwarf-Associated Picornavirus Encodes a 3C Serine Protease Recognizing Uncommon EPT/S Cleavage Sites
Source: Front Microbiol. 2021 Oct 13;12:757451. doi: 10.3389/fmicb.2021.757451 (PMC8549817; doi:10.3389/fmicb.2021.757451)
Supplement: Supplementary Figure 1 — Rice curl dwarf-associated picornavirus (RCDaV) was detected by RT-PCR in symptomatic rice and barnyard grass plants using RCDaV specific primers. [file Data_Sheet_1.zip › Table S5.DOCX]

**Table S5.** Pairwise sequence comparison of RCDaV with other viruses in the order *Picornavirales*.

Table S5A Pairwise amino acid sequence identities (%) of the P1, P2, P3, 3C and 3D proteins encoded by RCDaV compared to the representative members in the eight official families.

| **family** | **genus** | **species** | **Genome features** | | **Parwise amino acid identity(%)** | | | | | |
| --- | --- | --- | --- | --- | --- | --- | --- | --- | --- | --- |
|  |  |  | **GenBank accession no.** | **Size**  **(nt)** | **P1** | **P2** | **P3** | **3Cpro** | **3D** |  |
| *Caliciviridae* | *Vesivirus* | vesicular exanthema of swine virus | NC_002551 | 8284 | 23.6 | 19.9 | 22.2 | 25.9 | 23.7 |  |
| *Dicistroviridae* | *Aparavirus* | acute bee paralysis virus | NC_002548 | 9491 | 23.5 | 20.5 | 25.3 | 18.8 | 29.5 |  |
| *Iflaviridae* | *Iflavirus* | *Ectropis obliqua*  picorna-like virus | NC_005092 | 9394 | 19.8 | 20.2 | 23.3 | 19.1 | 23.6 |  |
| *Marnaviridae* | *Labyrnavirus* | *Aurantiochytrium* single-stranded RNA virus 01 | AB193726 | 9035 | 26.0 | 22.6 | 20.0 | 20.6 | 19.4 |  |
| *Picornaviridae* | *Aphthovirus* | foot-and-mouth disease virus | NC_004004 | 8134 | 20.7 | 26.3 | 25.8 | 22.2 | 28.1 |  |
| *Polycipiviridae* | *Sopolycivirus* | *Lasius neglectus* virus 1 | NC_035450 | 11851 | NA | 21.9 | 25.5 | 17.1 | 27.0 |  |
| *Secoviridae* | *Waikavirus* | rice tungro spherical virus | M95497 | 12226 | 23.5 | 22.1 | 27.4 | 23.7 | 28.9 |  |
| *Solinviviridae* | *Invictavirus* | *Solenopsis invicta* virus 3 | NC_012531 | 10411 | 20.4 | 18.0 | 21.8 | 16.4 | 25.0 |  |

Table S5B Pairwise nucleotide and amino acid sequences identities (%) of RCDaV compared with the seven unclassified picornaviruses. %N, nucleotide sequence identities (%); %A, amino acid sequence identities (%).

|  | **MaPV** | | **TUaPV1** | | **ApGlV1** | | **CVT** | | **HuPV51** | | **RBV** | | **LTaPV** | |
| --- | --- | --- | --- | --- | --- | --- | --- | --- | --- | --- | --- | --- | --- | --- |
| **Region** | **%N** | **%A** | **%N** | **%A** | **%N** | **%A** | **%N** | **%A** | **%N** | **%A** | **%N** | **%A** | **%N** | **%A** |
| Full length | 72.6 | - | 73.2 | - | 69.8 | - | 63.6 | - | 60.5 | - | 60.2 | - | 60.0 | - |
| 5’UTR | 87.7 | - | 82.6 | - | 79.1 | - | 66.7 | - | 63.1 | - | 62.9 | - | 61.8 | - |
| P1 | 75.6 | 82.0 | 72.6 | 78.6 | 74.5 | 81.2 | 65.9 | 59.9 | 63.8 | 49.1 | 61.9 | 44.9 | 62.3 | 44.8 |
| P2 | 67.2 | 63.3 | 71.0 | 73.7 | 66.5 | 64.1 | 60.9 | 43.2 | 59.7 | 27.3 | 59.9 | 26.5 | 60.3 | 27.3 |
| P3 | 73.1 | 77.5 | 74.9 | 80.2 | 69.3 | 73.1 | 63.9 | 57.9 | 58.7 | 39.9 | 60.6 | 40.8 | 59.8 | 40.4 |
| IGR | 52.4 | - | 71.8 | - | 58.4 | - | 68.1 | - | 64.1 | - | 63.9 | - | 62.5 | - |
| X1 | 59.5 | 31.0 | - | - | - | - | - | - | - | - | 60.1 | 23.1 | 54.3 | 22.7 |
| X2 | 66.8 | 64.3 | 56.2 | 48.6 | 61.4 | 47.1 | 43.8 | 20.9 | - | - | - | - | - | - |
| X3 | 71.5 | 69.7 | 56.7 | 52.8 | 62.1 | 49.7 | 54.6 | 28.7 | 60.9 | 14.6 | 63.7 | 18.6 | 61.5 | 19.7 |
| X4 | 63.5 | 57.7 | 73.4 | 76.9 | 70.2 | 75.0 | 61.1 | 41.4 | 56.4 | 22.9 | 67.0 | 19.6 | 65.2 | 21.7 |
| 2C | 68.5 | 68.2 | 77.3 | 86.3 | 66.5 | 70.0 | 61.9 | 50.5 | 58.2 | 31.8 | 56.8 | 35.1 | 58.0 | 32.3 |
| 3A | 57.3 | 40.9 | 64.5 | 63.6 | 59.8 | 41.1 | 56.9 | 25.0 | 56.9 | 20.5 | - | - | - | - |
| 3B | 62.3 | 65.2 | 69.6 | 73.9 | 68.1 | 73.9 | 75.4 | 78.3 | 66.0 | 36.8 | 57.6 | 21.7 | 57.6 | 21.7 |
| 3C | 70.0 | 73.6 | 72.5 | 75.2 | 70.1 | 76.8 | 61.2 | 56.3 | 57.6 | 35.1 | 57.1 | 27.8 | 57.2 | 30.5 |
| 3D | 78.4 | 87.9 | 78.6 | 86.6 | 71.3 | 79.5 | 66.0 | 65.0 | 59.3 | 46.1 | 60.9 | 46.3 | 59.6 | 44.3 |
| 3’UTR | 86.4 | - | 84.2 | - | - | - | 58.5 | - | 60.4 | - | 59.6 | - | 53.0 | - |
