# Supplementary figures and images for: A Novel Rice Curl Dwarf-Associated Picornavirus Encodes a 3C Serine Protease Recognizing Uncommon EPT/S Cleavage Sites
Source: Front Microbiol. 2021 Oct 13;12:757451. doi: 10.3389/fmicb.2021.757451 (PMC8549817; doi:10.3389/fmicb.2021.757451)

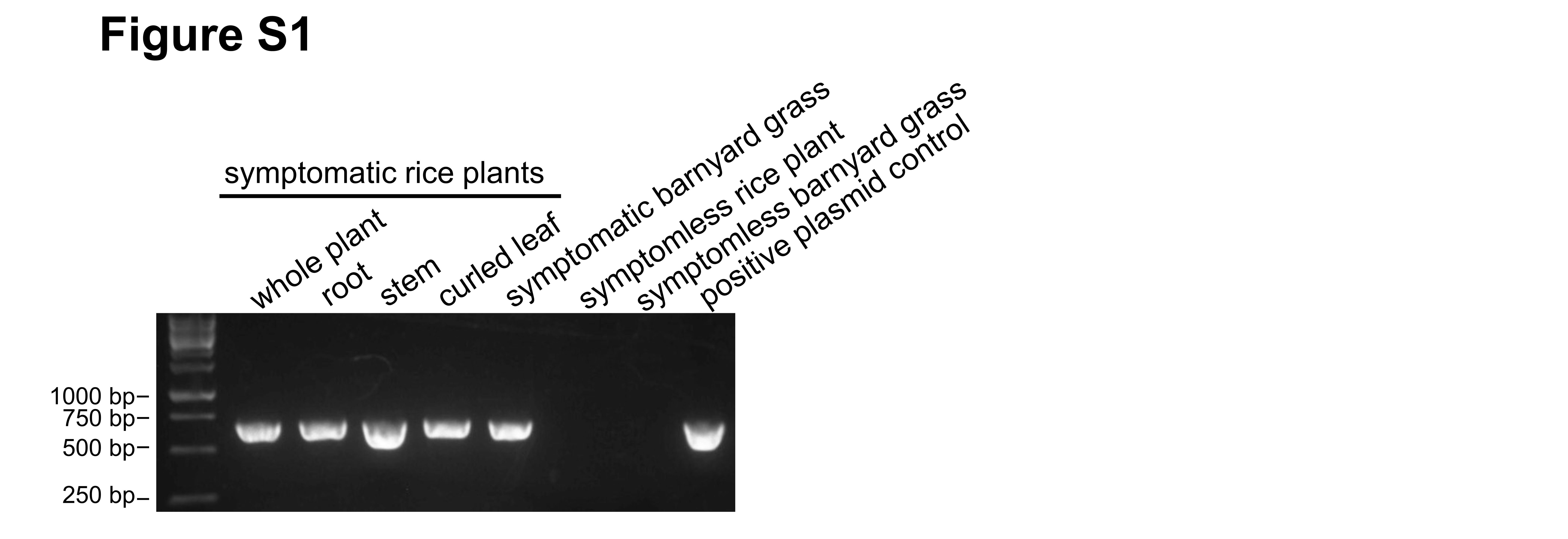

Supplement: Supplementary Figure 1 — Rice curl dwarf-associated picornavirus (RCDaV) was detected by RT-PCR in symptomatic rice and barnyard grass plants using RCDaV specific primers. [file Data_Sheet_1.zip › Figure S1.TIF]

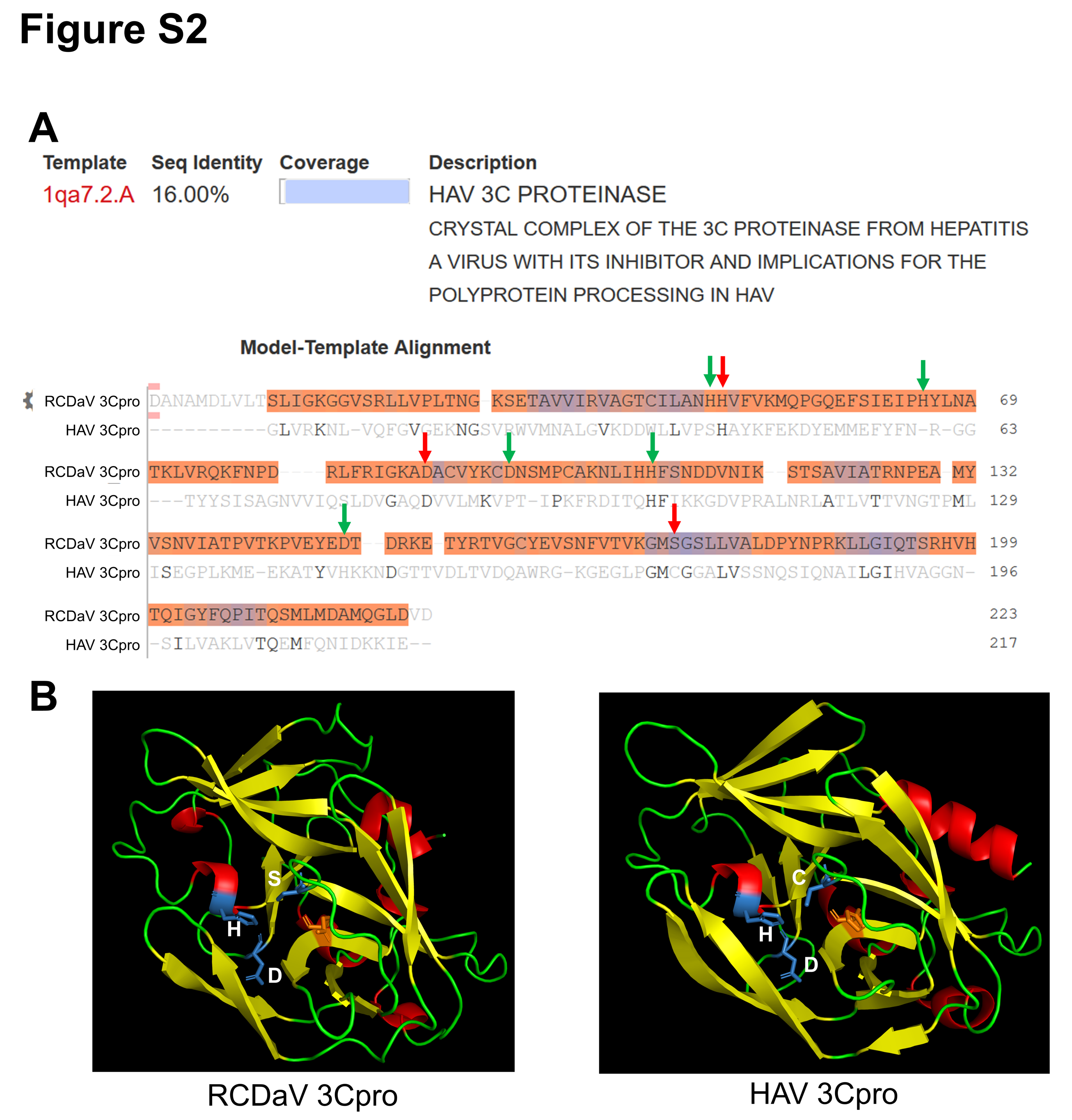

Supplement: Supplementary Figure 1 — Rice curl dwarf-associated picornavirus (RCDaV) was detected by RT-PCR in symptomatic rice and barnyard grass plants using RCDaV specific primers. [file Data_Sheet_1.zip › Figure S2.TIF]

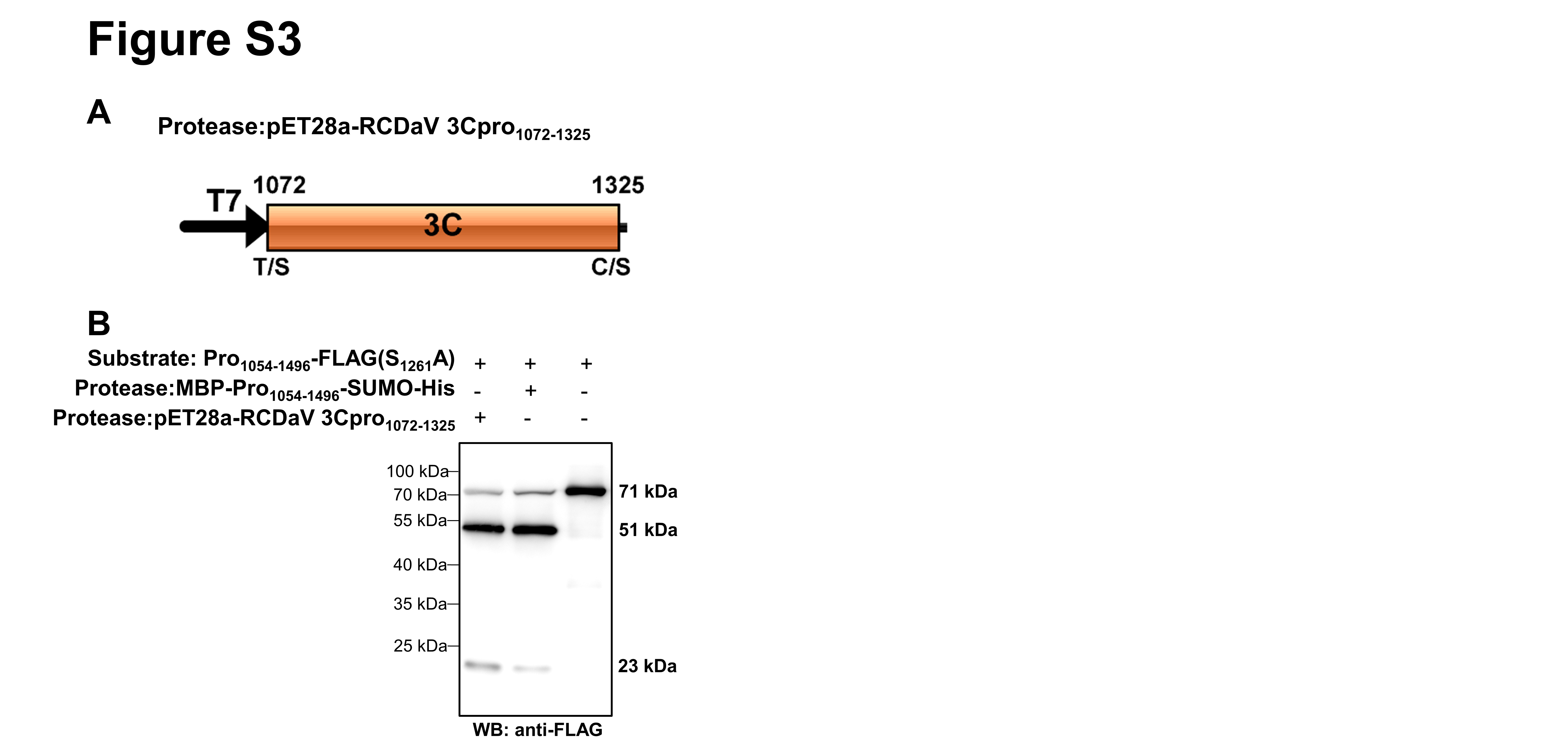

Supplement: Supplementary Figure 1 — Rice curl dwarf-associated picornavirus (RCDaV) was detected by RT-PCR in symptomatic rice and barnyard grass plants using RCDaV specific primers. [file Data_Sheet_1.zip › Figure S3.TIF]

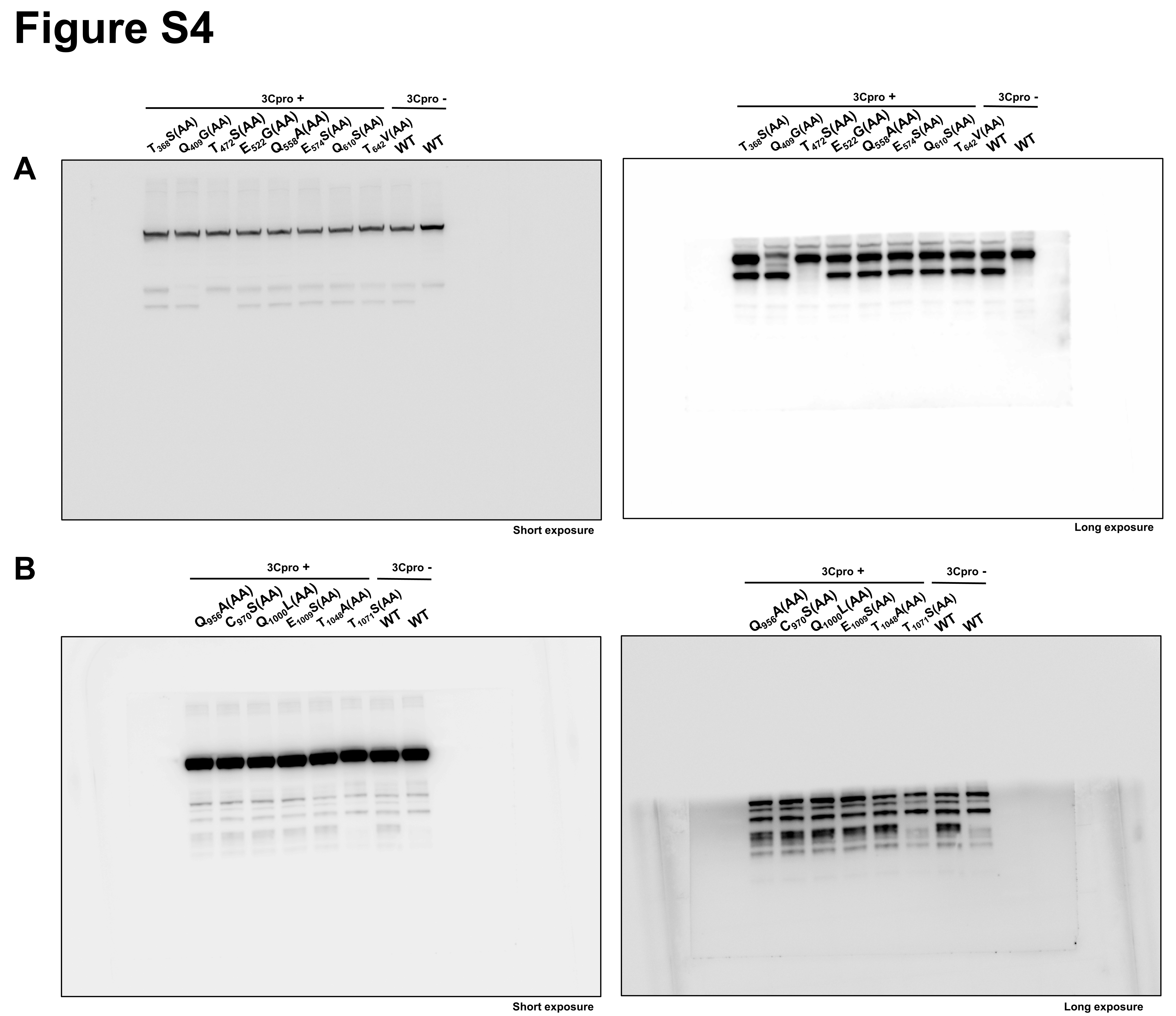

Supplement: Supplementary Figure 1 — Rice curl dwarf-associated picornavirus (RCDaV) was detected by RT-PCR in symptomatic rice and barnyard grass plants using RCDaV specific primers. [file Data_Sheet_1.zip › Figure S4.TIF]

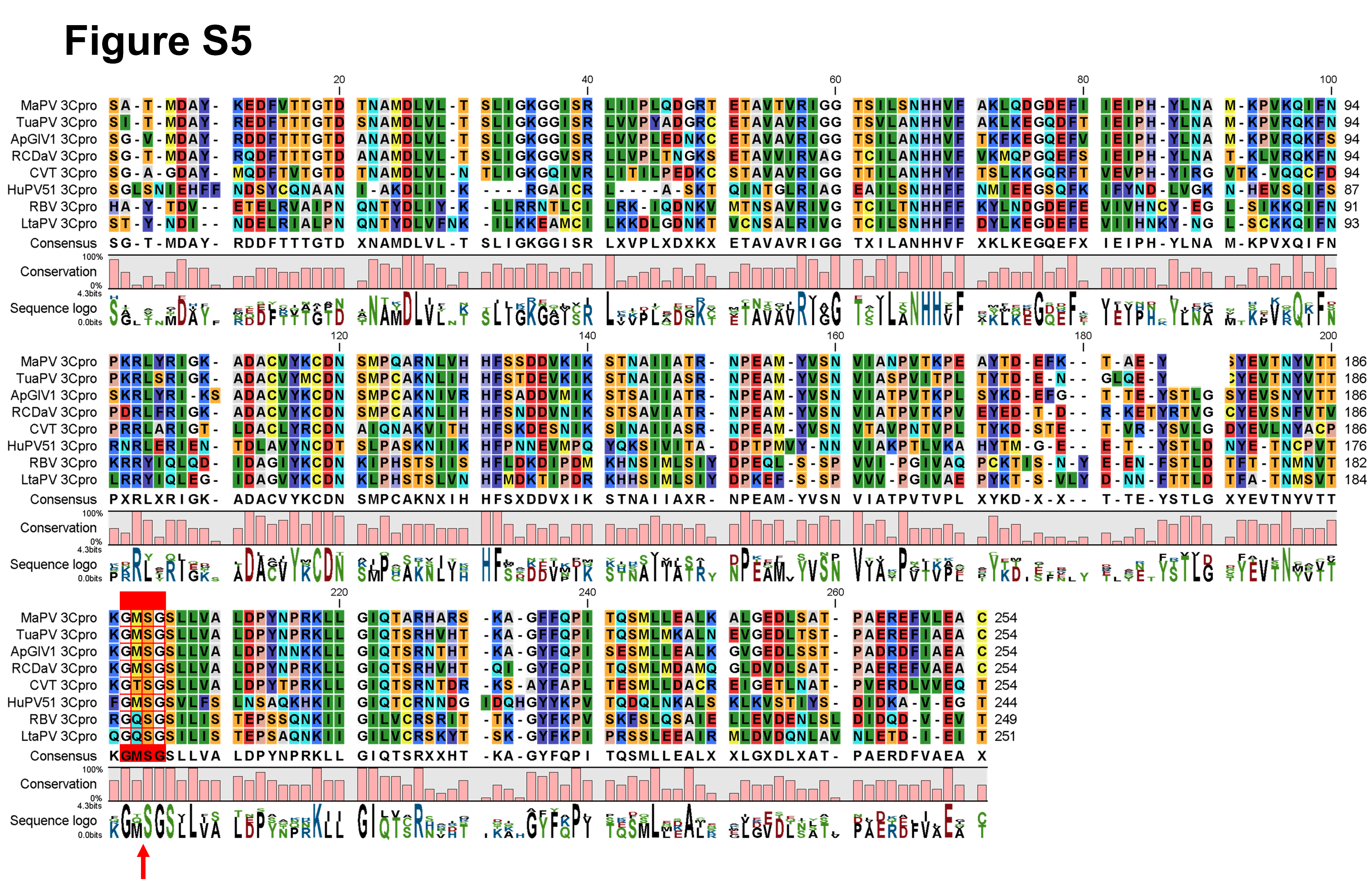

Supplement: Supplementary Figure 1 — Rice curl dwarf-associated picornavirus (RCDaV) was detected by RT-PCR in symptomatic rice and barnyard grass plants using RCDaV specific primers. [file Data_Sheet_1.zip › Figure S5.TIF]
